# Pediatric Pyomyositis: A Rare but Important Complication of Varicella

**DOI:** 10.1155/2020/3896264

**Published:** 2020-02-26

**Authors:** Luísa Vital, Luís Vieira, Bernardo Nunes, Frederico Raposo, Vitorino Veludo, André Pinho

**Affiliations:** ^1^Orthopedics and Traumatology Department, Centro Hospitalar Universitário São João, Porto, Portugal; ^2^Centro Hospitalar Universitário São João-Porto, Unidade Anatomia-DBM-Cintesis, Porto, Portugal

## Abstract

Varicella is a common viral infection in children and most of them recover without sequelae, but serious complications can follow this infection and 6% have been reported to be musculoskeletal. A previously healthy 3-year-old Caucasian male presented with odynophagia, anorexy, fever, refusal to bear weight, and vesicular exanthema. Varicella was diagnosed, but he sustained fever around 39°C and local tenderness on the proximal lateral portion of the right leg maintaining an antalgic position. Ultrasonography and plain radiography were performed, but the magnetic resonance imaging (MRI) was performed due to the clinically worsening diagnosed pyomyositis. He was subjected to surgical debridement, and we started intravenous antibiotherapy. *Streptococcus pyogenes* grew in the microbiologic culture. At a 6-month follow-up, the boy did not suffer from any sequelae. The regular course of varicella is benign; however, it can occasionally develop into a more serious illness. The initial presentation of pyomyositis is often subacute, and the first symptoms may be vague. The awareness of musculoskeletal complications is imperative, and the combination of varicella's exanthema and fever followed by some limb complaint should lead to an alert attitude.

## 1. Introduction

Varicella is a common viral infection in children, and most of them recover without sequelae. Serious complications following varicella zoster virus infections are uncommon and reported to be approximately 2% [1]. Of all complications, 6% have been reported to be musculoskeletal and could be life-threatening or limb-threatening [2, 3].

*Streptococcus pyogenes* (group A streptococcus) is a major human pathogen and responsible for a wide range of both suppurative and nonsuppurative diseases worldwide, for example, pharyngitis, erysipelas, septicaemia, meningitis, pneumonia, and the notably severe manifestations necrotizing fasciitis and streptococcal toxic shock syndrome (STSS) [4]. In cases of diagnosed varicella, concerning secondary bacterial complications, *S. pyogenes* has been described to be the leading cause and to be the major risk factor for invasive disease with an unfavorable long-term outcome [5]. Among children, varicella is the most important risk factor identified for the acquisition of group A streptococcus (GAS) infection [6].

## 2. Case Report

A previously healthy 3-year-old Caucasian male presented to his local hospital emergency department with odynophagia, anorexy, fever, refusal to bear weight, and vesicular exanthema. After analytic study and hemocultures, he was transferred to a central hospital due to his orthopaedic complaints. His blood test revealed a total white blood cell count of 14.44 × 10^9^/L and C-reactive protein (CRP) of 118.2 mg/L. One day after admission, sustained by the exanthema evolution, he was diagnosed with varicella and the ibuprofen intake was suspended. Because of persistent refusal to bear weight and assumption of antalgic position with the flexed right knee, a hip and knee ultrasonography was performed which did not reveal any alteration as well as lumbar and hip plain radiography that was also normal. The boy sustained fever around 39°C and worsening of local tenderness on the proximal lateral portion of the right leg maintaining an antalgic position.

Supposing it was a cellulitis, he initiated empiric antibiotherapy with flucloxacillin and repeated ultrasonography which showed prominence and augmented echogenicity of the superior anterolateral muscular plane of the right leg that suggested myositis. At this time, his lab testing for creatine phosphokinase (CPK) and myoglobin was normal but still showed leukocytosis and high CRP.

**Figure 1:**
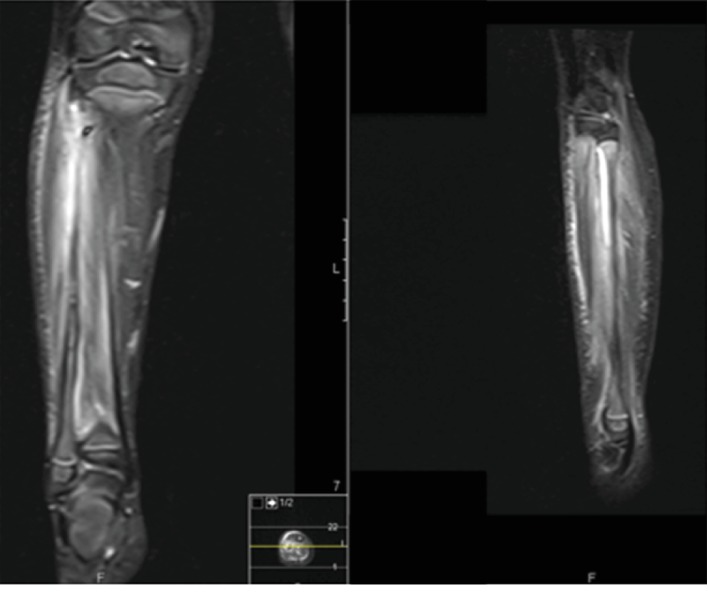
MRI (T1 STIR) of the left leg showing abscess involving the adjacent muscle and fascia.

Due to increased pain complaints and leg inflammatory signs, associated with persistent fever and CRP consistently above 100, magnetic resonance imaging (MRI) was performed, which revealed an extensive infectious process involving the upper half of the fibula with periosteum detachment as well as a 6.1 cm abscess involving the adjacent muscle and fascia (Figure 1).

He was taken to the operating room (OR) where surgical debridement was performed and a sample of pus was collected and sent to microbiology. Intraoperatively, the absence of articular involvement was confirmed.

Antibiotherapy with clindamycin was added. *Streptococcus pyogenes* grew in the microbiologic culture of the pus surgically obtained. *Streptococcus pyogenes* was also isolated from the blood culture previously collected.

Three days postoperatively, he revealed significant clinical and analytical improvement. He was discharged after completing 15 days of intravenous antibiotherapy and had no gait complaints. After being discharged, he was maintained on 15 days of oral antibiotic, and at 6 months of follow-up, the patient did not present any complaint or sequelae.

## 3. Discussion

Varicella is an acute contagious disease that usually affects children. The regular course of the disease is benign; however, it can occasionally develop into a more serious illness. Varicella-related musculoskeletal infections are rare but can cause significant morbidity or mortality. Recent patterns suggest an increase in the incidence during the last decade [7]. To our knowledge, no case of pyomyositis of the leg has been described as a complication of varicella.

Pyomyositis is a bacterial infection of the skeletal muscle and often results in localized abscess formation, and although the most common organism identified is the *S. aureus*, this infection which is related to varicella usually involves GAS [8]. The probable mechanism of group A streptococcus in varicella has been related to the genetic predisposing factors and a possible link to the nonsteroidal anti-inflammatory drugs [9]. Our case shows an example of delay in varicella diagnosis that led to the use of these drugs, which may have been a factor of clinical deterioration.

The initial presentation of pyomyositis is often subacute, and initial symptoms may be vague. Three stages of the disease are described. The invasive stage is characterized by low-grade fevers, general malaise, and dull, cramping pain. Although the overlying skin often appears normal, a firm texture may be appreciated on deep palpation. Abscess formation occurs during the suppurative stage, and patients tend to have more focal complaints. Often, there is increased tenderness with overlying erythema and swelling. During the late stage of pyomyositis, patients develop high fevers, exhibit more local signs of infection, and complain of severe pain. Patients in the late stage of pyomyositis can develop systemic manifestations, including metastatic abscesses, arthritis, and renal failure. Septic shock and toxic shock may ensue if urgent management is not initiated [10–13].

Normally, there is a time interval between the onset of the varicella rash and the first symptom or sign of a related complication. In this case, apparently, they started at the same time, which is not in concordance with other reported cases and may have been a confounding factor in the course of the disease.

In diagnosis, although laboratory studies tend to be nonspecific, the complete blood count usually shows leukocytosis, and there are elevated CRP levels and erythrocyte sedimentation. Radiologic imaging revealing muscle inflammation is necessary for the definitive diagnosis, and imaging options include ultrasound—which is able to show the site, size, and type of fluid in the area of collection—and CT scanning and MRI—which are reported to be more specific in showing associated bone or joint involvement but may require anesthesia in children and may not be available in emergency situations [14]. Culture of blood and muscle aspirate, whenever possible, is mandatory.

Because GAS infections complicating varicella range from cellulitis, abscess, and septic arthritis to life-threatening necrotizing fasciitis and pyomyositis, the delay in diagnosis should be avoided, and in the absence of clinical markers, a high index of suspicion is required with any child with varicella who refuses to bear weight or has joint pain and elevated CRP levels in the blood [7, 14].

The majority of varicella-related musculoskeletal infections, usually caused by group A streptococcus, require both antibiotic treatment and surgical debridement. The two main antibiotic treatment options are penicillin and clindamycin. While penicillin may be less efficacious in the treatment of group A streptococcus pyomyositis because of the large bacterial load which consists predominantly of nonreplicating cells by the time of diagnosis, clindamycin may have better efficacy because it is less affected by inoculum size or treatment delay and it suppresses toxin production [15]. However, in contrast to other streptococci, *S. pyogenes* has to date remained universally susceptible to penicillin [4]. Antiviral medication, like acyclovir, is effective at early stages of varicella but has no apparent influence on complications [16]. Other options to treat severe infections caused by GAS are being developed, as an intravenous polyspecific immunoglobulin contains neutralizing antibodies against a wide spectrum of streptococcal superantigens and appears to decrease mortality in severe infections [17].

## 4. Conclusion

Varicella is a frequent disease affecting children and, although usually self-limited, could be associated with infrequent but serious complications. The awareness of musculoskeletal complications is imperative to any physician who evaluates these children, and the combination of varicella's exanthema and fever followed by some limb complaints should lead to an alert attitude.
